# Intelligibility in microbial complex systems: Wittgenstein and the score of life

**DOI:** 10.3389/fcimb.2012.00088

**Published:** 2012-07-12

**Authors:** Fernando Baquero, Andrés Moya

**Affiliations:** ^1^Department of Microbiology, IRYCIS, Ramón y Cajal University Hospital, and Division of Extremophily and Evolutionary Biology, Centre for Astrobiology, INTA-CSICMadrid, Spain; ^2^Network Research Center for Epidemiology and Public Health (CIBERESP)Madrid, Spain; ^3^Cavanilles Institute on Biodiversity and Evolutionary Biology, Genomics and Health Area, Centre for Public Health Research, University of Valencia, Conselleria de SanitatValencia, Spain

**Keywords:** intelligibility, complex systems, Wittgenstein, metaphors, epistemology

## Abstract

Knowledge in microbiology is reaching an extreme level of diversification and complexity, which paradoxically results in a strong reduction in the intelligibility of microbial life. In our days, the “score of life” metaphor is more accurate to express the complexity of living systems than the classic “book of life.” Music and life can be represented at lower hierarchical levels by music scores and genomic sequences, and such representations have a generational influence in the reproduction of music and life. If music can be considered as a representation of life, such representation remains as unthinkable as life itself. The analysis of scores and genomic sequences might provide mechanistic, phylogenetic, and evolutionary insights into music and life, but not about their real dynamics and nature, which is still maintained unthinkable, as was proposed by Wittgenstein. As complex systems, life or music is composed by thinkable and only showable parts, and a strategy of half-thinking, half-seeing is needed to expand knowledge. Complex models for complex systems, based on experiences on trans-hierarchical integrations, should be developed in order to provide a mixture of legibility and imageability of biological processes, which should lead to higher levels of intelligibility of microbial life.

## Introduction

Life is a highly complex system, including the most complex objects in the known universe (Bedau, 1996). The genomics revolution has catapulted molecular biology, and particularly microbiology (Westerhoff and Palsson, 2004), into the realms of systems biology approaches to complex systems. Such a trend was based on the growing compelling intuition of the need of scaling-up molecular biology, in a new age of synthesis requiring formal integrative tools (Baquero, 2004, 2009). Biochemistry and lately Molecular Biology have shown that certain distinctive carbon-based macromolecules play a crucial role in the vital processes of all known living entities, but life seems to be more in the nature of a process (Bedau, 1996). The epistemological problem is how to cross the gap transitions between successive levels of understanding that corresponds to the different hierarchical levels of the complex system of life. Microbiologists are the best positioned scientists to respond to such a challenge, as they are familiar with “multiple-levels biology,” dealing simultaneously with microbial collectives and collective genomes, as in metagenomics (Moya et al., 2012), cell-to-cell interactions (including pathogenesis, Desnues et al., 2010), the flowing biology of sub-cellular mobile genetic elements (Beiko et al., 2005) and finally with the wealth of gene-gene epistatic interactions (Babu et al., 2009).

Microbial communities, species, clones, plasmids, transposons, integrons, and genes are evolutionary individuals tracing their evolutionary trajectories at different hierarchical levels (Baquero, 2011). Such trans-hierarchical network-like complexity simply eliminates the possibility of identification of simple causal structures, if they ever exist out of our ways of representation (Schrodinger, 1957). The hope of a simple answer to the classic Baconian question in science “What is the cause of … ” has no sense any more, and in fact the complex structure of biological processes constitute the major challenge for Biological Theory (Callebaut and Laubichler, 2007).

The challenge is not only to deal with quantitative integration of elements across these major hierarchical transitions in microbiology, or in biology at large (Maynard Smith and Szathmáry, 1997), but to eventually discover general principles of microbial life rather of just keep on descriptions (Westerhoff and Palsson, 2004). Such scaling-up process of understanding resembles the escalation from the *forms* of a language (as lexical or syntactic) toward its *meaning* (semantics) (Steels, 2004, 2010; Rosen, 2004). Interestingly, the trade-off between these hierarchical levels in a shared world has been defined as *intelligibility* in linguistic theory (Komarova and Niyogi, 2004). In his primary sense, the word *intelligibility* reflects the possibility of such a trans-hierarchical understanding. St. Thomas even derives the Latin word *intelligere* from *intus legere*, or “reading into”; even if the origin were *inter legere*, the term stresses the need of reading beyond the words and sentences, in another cognitive dimension, to reach the meaning. Accordingly to the Cato's classic sentence, “*legere, et non intelligere, neglegere est*,” that is, “as good not read, as not to understand.” In this work, we use the word “*intelligibility*” as the construction of meaningful (thinkable) models in response to the assimilation of knowledge (clear-and-distinct or fuzzy), and able to reflect to a certain extent the reality of complex natural systems, as those which are the objects of biological sciences. The conversion of data into knowledge constitutes a great challenge for future biological research (Brenner, 2010). In fact intelligibility is a prerequisite to developing modern biology grounded on a sound epistemology (Dougherty and Bittner, 2010). “*Legere, et non intelligere, neglegere est*”. Two thousand years after Cato, Albert Einstein formulated essentially the same idea: “*Science without epistemology is—insofar as it is thinkable at all—primitive and muddled*” (Einstein, 1949).

Metaphors are frequently used by scientists as “non-logic” epistemological aids to think about reality (De Man, 1978). Biologists have long made use of linguistic metaphors in describing and naming cellular processes, and in particular involving from DNA as language to genome as a “book of life.” The current questions are: (1) if these apparently immediate analogies might result in a deeper possibility of analysis of genetic-genomic structures using methods that have been developed in linguistic research; and (2) if such an analysis will enable to understand the general principles and processes of life, and even (not entering here again in the universal's problem) life itself as a intelligible entity.

The image of genome as a “book of life” has attracted popular imagination, but it is obvious that the knowledge of the entire genomic sequence of *Haemophilus influenzae* (Fleischmann et al., 1995) or *Homo sapiens* (McPherson et al., 2001; International Human Genome Sequencing Con-sortium, 2004) has not resulted in a much deeper understanding of the “life” clues of these organisms. This occurs not only because of our gaps in understanding the function of all genes and the complexities of regulatory and epigenetic interactions between genes and other meaningful sequences. Probably the human way of reading a text is simpler than the cell way of reading. Human language texts are read in one way only, sequentially and involving all characters. Genetic texts are “read” by cellular mechanisms in several different ways, each time using a different selection of the characters of the same text while skipping others. Indeed the “score of life” could be a better image of the genomic language. A score is a series of staves on which all the different instrumental and/or vocal parts of a musical work are written, one under the other in vertical alignment, so that the parts may be read simultaneously.

But in this report we consider there exist even bigger difficulties to predict how relevant is deciphering the language of genes, the “book of life.” One key epistemological problem is to discuss about our ability to clarify the possible relations between the structure of a possible language (genetic and genomic sequences) and the characteristics of life of particular organisms, which seem to be determined by this language. Obviously this is a problem of reductionism (Wimsatt, 1976)—might the understanding of life be reduced to the understanding of the genetic-genomic language?

In the way of thinking of Ludwig Wittgenstein, and even more sharply in his friend and commenter, Moritz Schlick (Schlick, 1936), the meaning of the word “life” can only be *shown*, not understood and consequently not *clearly* expressed in propositions. It might sound paradoxical to attach to this statement in the age of glory of genomics, proteomics, and metabolomics. Life is a *fact* that can be *shown* (but not defined by) as something like a moving and loosely integrated complex of contingent structures, each one of them (and the complex itself) tending to be sequentially replaced by similar forms, and displaying various degrees of changes in variability and complexity both during almost instantaneous and long-term periods of time. Note that because we are here only *showing* life, this description does not assure that we are not confronted with non-living structures with similar properties, and certainly that any notion of progress or purposiveness cannot be considered here. Nevertheless, as the human *observers*, we are not neutral in the process of selecting what we would like to *show*, as frequently we are confronted with a non-descriptible feeling of sharing a common quality (“animation?”) with what we tend to show as living things.

## The “score of life” metaphor

The Ludwig Wittgenstein's “*Tractatus logico-philosophicus*,” published in English in 1922 under the guidance of Bertrand Russell, is widely recognized as one of the main post-kantian approaches devoted to explore the possibilities of human knowledge of natural world (Wittgenstein, 1921). In its theorem 4.0141, Wittgenstein compares music scores and gramophone (DVDs, in our times) with music.

4.0141

In the fact that there is a general rule by which the musician is able to read the symphony out of the score, and that there is a rule by which one could reconstruct the symphony from the line on a gramophone record and from this again—by means of the first rule—construct the score, herein lies the internal similarity between these things which at first sight seem to be entirely different. And the rule is the law of projection which projects the symphony into the language of the musical score. It is the rule of translation of this language into the language of the gramophone record.

The order and qualities of the musical notes in the score, the grooves' irregularities in the gramophone record, in summary, the “language” from which music might be reproduced, is not music, but has an *internal similarity* with music. Much longer before the discovery of the genetic code, the Wittgenstein's theorem 4.0141 recalls the main structural feature of living organisms. The process of reading the score (genetic language), produces music (life); conversely, music can be converted, translated, by a “law of projection” into a musical score, and from this again music might be reconstructed. Without internal similarities, these transitions between series of objects “that at first sight seem to be entirely different” should be simply impossible.

Interestingly in music, as in life, the description of “what is said” beyond the individual sounds is obscure. As the classic question of Erwin Schrodinger what is Life? The question: what is Music? refuses precise answers. No propositions are transmitted by music to describe clear and distinct facts, able to be thought (logically considered) by human mind. There is, as in genetics, a certain “arithmetic order” of notes that is required to produce obscure final effects. In Leibnitz words: “*exercitium arithmeticae occultum nesciendis se numere animi*” (*Leibnitii epistolae, collectio* Kortholdi, ep. 154), that is, music is as an unconscious arithmetic's exercise in which mind do not know what is being counted.

Maybe one of the difficulties of thinking life using linguistic structures is the fluid, dynamic nature of life. Languages, music score or genetic-genomic sequences, are essentially static. A book, or a musical score, or a genome sequence can be indefinitely stored without any alteration, and even more, without producing any effect (except covering a small parcel of physical space). On the contrary, speech, music, or life, are essentially dynamic; without movement they ceases existing. The fact that linguistic structures “contain” potential dynamicity does not make them dynamic at all; indeed they are practically nothing by themselves. The key-fact is that between languages and dynamic phenomena an *interpretative intermediary* should be interposed. The music score gives rise to music only if interpreters are available, musicians (four in a string quartet) able to read the language and converting it into sounds. Indeed a music score has an ordered internal structure, for instance following the rules of harmony, but, at first sight, we could conclude that in the absence of correct interpretation, a music score cannot be differentiated from a random sequence of notes.

Let us now imagine an out-of-Earth scientist examining a music score. He has no idea about notes, instruments, or sounds, even about the existence of music at all. Probably he will be able to differentiate a music score from a random sequence of notes. Some notations (notes) are preferentially linked to other ones, some conserved and iterated sequences are recognizable, the role of black and white notes seems not identical, some occur more frequently than others when accompanying the name a particular instrument (unknown). The note's frequency per decimeter of score apparently depends on some mysterious words at the margin as “*Andante scherzoso quasi alegretto*,” that nevertheless might provide a “living equivalent.” He could conclude that the musical score has a linguistic structure, potentially leading to an unknown type of dynamic behavior. If the out-of-Earth scientist could had access to a high number of different scores, he could even trace different schools, authors, influences, even a history of this unknown language—and probably he will not be much far from reality. In summary, an analytical “science” of this language could be built, and that in the total absence of knowledge about the nature of music.

Now note that the mirror process of analysis is also possible. In that case our second out-of-Earth scientist is observing the performance of a music group playing the Schubert's Piano Trio in B flat, D. 898. Unfortunately, he does not know about the existence of music, as he is unable to hear any sound, but he is able to distinguish the keys, bows, and strings of the different instruments and he can precisely record any movement of the player's arms and fingers on these structures. A representation of these movements during time should produce something similar to the musical score of the Piano Trio. Indeed the precise record of these movements might substitute the musical score, and when applied to the instruments should reproduce the music. As in the previous case, a collection of this type of records could lead to tracing schools or authors, or a history, or even a science of this language—without knowing what music is at all.

But we can also conceive a third out-of-Earth scientist, able to hear the sounds and to correlate them with the instruments and the movements of the players. It might well happen that the scientist could perceive the separate sounds, but he is either unable to link them in his mind as significant ensembles (melodies), or the sounds are so different in his brain than in ours, that our harmony is totally useless for his sensibility, and out of any esthetical possibility. As in the previous cases, this scientist could be able to study the history of music, without understanding at all what music is.

The essential is to discuss if a particular sequence of written musical notations, or sounds, or hands and finger movements, has only the meaning of “music” when understood by a particular type of sensibility. Even more: we can replace the “out-of-Earth” scientists by musicians, which will be able to reproduce the music without knowing anything about its nature, and without experimenting any of the effects that music might cause in the appropriate sensibility. They are in a “Chinese room” situation, in which the (considered to be intelligent) intermediate within the closed room receives below the door messages in an unknown language, but accordingly to a set of rules, he is able to produce responses in the same unknown language (Searle, 1984). It is obvious that the music score, or the genome sequence, is totally unaware of its function in the process of life, and the same is true for other possible intermediaries, for instance, involved in protein translation. In the words of Sydney Brenner, “genomes do not contain in any explicit form anything at a higher level than genes” (Brenner, 1999), or, paraphrasing Leibnitz when describing monads, “genomes do not have windows.”

Therefore, neither from the outside, in which life can only be *shown* (and even that, without certitude), nor from the inside (life is invisible for life-determining structures), life seems to be thinkable. “We feel that even if *all possible* scientific questions be answered, the problems of life have still not been touched at all. Of course there is then no question left and just this is the answer” (Theorem 6.52). In other words, the answer is that to ask ourselves for the meaning of life is a false question, that is, there is nothing to think about. “For an answer which cannot be expressed the question too cannot be expressed” (6.5). And, as stated in the last sentence of the *Tractatus*, “Where of one cannot speak, thereof one must be silent” (6.54). The main interest of the “score of life” metaphor is probably that both life and music can be *shown* (as something that seems to impose a reality), *but not thought* (we cannot say anything about its reality), as the genomic sequences or the musical scores are mere representations of these obscure realities.

## Thinkability of life

If life can be only *shown*, is Ludwig Wittgenstein right? Have the terms “*Bio*logy” and their derivatives, as “Micro*bio*logy,” intrinsic epistemological contradictions? Are they non-sense proposals? We arrive now to an apparent contradiction. On one hand, we could reach the notion that life is unthinkable. But, as stated in 3.02, “the though contains the possibility of the state of affairs which it thinks. What is thinkable is also possible.” On the other hand, life is *perceived* as a *fact*, therefore not only possible, but a realized entity. Obviously, if it exists, it should be thinkable. If life is not thinkable, either life does not exist, or has an unveiled, hidden reality. This antinomy is a clear variation of the Kantian ones, based on the confusion between the spheres of *phenomena* and *noumena*, and encapsulates the main problem that is discussed in this assay. Life is perceived, even *experienced* as a fact, but it is not a fact, it is not an entity. If that proposition were true, life should not be an object of natural science. Limiting the thinkable and thereby the unthinkable, philosophy limits the disputable sphere of natural science (Wittgenstein again, see 4.113–4.114).

Natural science should be thinkable and speakable. Everything that can be thought at all can be thought clearly. Everything that can be said can be said clearly (4.116). If life is unthinkable, but if it had a reality, we should think on “pictures of life” using articulated propositions, which are “models of reality as we think it is” (4.01). “The proposition constructs a world with the help of a logical scaffolding, and therefore one can actually see in the proposition all the logical features possessed by reality *if* it is true” (4.023). Wittgenstein's propositions could be considered as something derived from the combinatorial and ordered nature of structures as the musical score (3.141), or, in the life context genomes, again “representing” the (suspected) reality, but *unable to represent what they have in common* with reality (4.12). The gap is maintained between the unthinkable but presumed reality (life) and the thinkable picture of it (proposition).

Life is as thinkable as music is thinkable. In both cases, there is what Wittgenstein calls a certain “experience of meaning.” Understanding life and music is to perceive “fine shades” of meaning. Intuitively both music and life seems to be meaningful, but in both cases they seem to be resistant to any “semantic” treatment. It has always been difficult to see how “meaning” could be fruitfully ascribed to music, as this notion is applied to language (Bar-Elli, 2006). Obviously the understanding of music has nothing to do with the ability to recreate in mind a memorized melody, or to foresee in the concert hall the next variation of a musical theme. We can say the same for life. We can predict, with a certain confidence, what will happen in the next step, based on our experiences, but that is not to understand—and even not to think about life. Experiences might provide a flavor of causality, following Hume, regularities in structured observations leads to expectation (Dougherty and Bittner, 2010), but that is not real understanding. We are just following something that we can only *show*, in a sense, as the conductor of the orchestra is showing with its baton, a kind of mixture of performance and unthinkable matter.

This simultaneous experimentability and unthinkability of music was analyzed in detail by Arthur Schopenhauer, in one of the chapters of his seminal book “*The World as Will and Representation*” (Schopenhauer, 1958). He stated that the music, ignores the world of concrete phenomena, and therefore only *resembles* some original reality than cannot be copied. Schopenhauer believes that music resembles, represents, “is a copy of the *will itself*, the objectivity of which are the Ideas.” He will underline after some paragraphs his convergence with Leibniz. As stated before, “*Musica est exercitium metaphysics occultum nesciendis se philosophari animi*,” that is, music is an unconscious exercise of metaphysics where the mind does not know what is thinking about. Of course Schopenhauer is a vitalist, and biologists will immediately recognize here the relation between *will* and the obscure dynamics of life, and might immediately reach the intuition that the *will* of music might be a copy, a representation of the *will* of life. The effects of music on humans could be derived from the *recognition* (let us accept here this platonic term!) of something common between our obscure perception of life and the music itself. Reinterpreting Schopenhauer, the *will* represented in music is a representation of the *will* of life, that is, a common *will* is independently perceived (can be *shown*) in both the music and life. In principle, we cannot speak about representations between two entities of the same hierarchical order, in our case, two equally unthinkable entities. It could be suggested that, even among unthinkable entities, there may also exist a hierarchy, so that entities that are lower in the hierarchy (music) might be able to represent higher ones (life).

## Major transitions: a trans-hierarchical cycle of representations and represented entities

Between the groove pattern and the music there is a “major transition,” essentially a qualitative transition. Similarly, between the genes and the life of a bacterial cell, or between cells and the complex living expressions of an ecologically-integrated community of cells, there are major transitions, as those identified by Maynard Smith and Szathmàry in evolutionary processes (Maynard Smith and Szathmáry, 1997). In both cases, there is a collection of “small” parts that assemble to produce qualitative different larger wholes. It is obviously tempting to propose that the lower levels of the hierarchy blindly “represent” the higher levels, as the way they are assembled (its order) has something formal, a correspondence, with the higher level activities. Evolutionary biologists will be prone to accept that there are “levels of life” as the development of life seems to occur from single replicating molecules, which provided the bases for reproduction at higher levels, as cells or organisms, in a successive series of “major evolutionary transitions.” But it will be difficult to accept that “lower levels” will be able to “represent” the higher levels. That occurs because, if there is an general evolutionary flow from the lower to the higher hierarchical levels, the different entities at the higher levels of life categorically imposes particular organizations of lower levels. Indeed processes as speciation depends on the imperatives of higher over lower hierarchical levels.

The continuous interplay between hierarchical levels is a trade-mark of life (Campbell, 1974). Some kind of unity based on reciprocal trans-hierarchical effects occurs there between what is represented and the representation itself. Indeed it is easy to imagine that the life of a particular bacterial organism is to a certain extent represented in the organization of ifs genome, as the music of a particular Mozart's string-quartet is represented in the organization of its musical score. But note that in both cases the representation is a “generational representation” for the represented entity, that is, the re-production of a particular bacterial organism or string quartet is entirely dependent from the representation, the genome, or the score, respectively. On the other hand, the score is meaningless in producing music in the absence of an instrument. In modern times (the origin of life might be another case), the genetic sequences (the representation) are only *meaningful* if a specific living system (what is represented) is present. The meaning of the representation should be perceived by the living system, which produces a biological scaffold (an instrument) at its turn generates its own representation, what is required for reproduction. The higher hierarchical level (the living system) has the lead in the process, as there is no representation without anything to be represented. That fits with the common wisdom in biology: the content and order of sequences in the genome corresponds to what has been selected by life itself, the complex and dynamic living interplay between the cell and its environment. For instance, speciation requires the dominance of “what it is represented,” the adapted phenotype, over a particular genome. Essentially genetic plasticity and modularity expresses such subordination. We found here an interesting trans-hierarchical cyclic correlation between representation and reproduction, a correlation that is produced blindly in both senses, as in the metaphor of the messages crossing a Chinese room (see above). Indeed we suspect that in ancient evolutionary times there was no difference between what was represented and the representation itself, the evolution of life consisting in digging a “major hierarchical transition” between both entities.

How that model applies for the music score? We should admit that the score, being the representation of music, produces music only if there is an instrument that converts notes into sounds, and only if there is somebody with an “experiment, in a sense, to understand (even in an unthinkable way) the *meaning* of the music. Because of its experiential capacity, and the derived effects of such kind of understanding, the human being listening music (higher in the hierarchy) is able to produce suitable instruments for reproducing again something *from himself*, perhaps the obscure *will* of Schopenhauer. The metaphor resists: the music score represents something unthinkable, this will, and the subject of the will (life in general, or a human mind) produces representations to perpetuate the re-presence of the effects associated with the will.

## Thinking on representations

The major task of science is to explore and expand the limits of intelligibility. The main current task of biology is to understand the correspondences between genomes and life. (Lewontin, 1974; Ferrada and Wagner, 2012; Wagner, 2012a,b). A representation is based on *correspondences* between what is represented and the representation itself. The representation cannot be at the same hierarchical level than that what is being represented. What might be the correspondences between life or music, and the artifactual representation of these presumed realities, as genomic sequences or musical scores? Let us go back to the *Tractatus*. “It is clear that, however, different from the real one an imagined world may be, it must have something—a form—in common with the real world” (2.022). What it is common are the *forms*. “We make (our logic, our language) to ourselves pictures of facts” (2.1). This picture is a representation of the facts, in which the elements of the picture correspond to the objects (2.13), linked in a definite way accordingly to what it is imposed by their forms (2.14), and logically indicates the possible non-existence of some facts (2.11). The picture, the representation of the reality, is in itself a fact (2.141), “as the elements of the picture are combined with another in a definite way, representing that real things are so combined with another” (2.15; 2.15.14). These *co-ordinations* are as it were the feelers of its elements with which the picture touches reality (2.1515). “What the picture must have in common with reality in order to be able to represent it after is manner—rightly or falsely—is its form of representation” (2.17). In summary, “the picture has the logical form of representation in common what it pictures” (2.2). All that certainly has a certain platonic flavor, as the picture, the representation, can be something made by drawing the shadow of the reality on our mind's screen. Biology is the art and science of finding the *forms* able to *represent* life.

As any other type of knowledge, biology should be based on propositions. Only propositions have sense; only in the context of a proposition has a name meaning (3.3). Propositions do not have meaning, but sense, reflecting all possible situations they represent. Only the relations, the order matter, not the things, not the objects themselves. A proposition is the description of a fact (4.023), a fact that involves objects. The possibility of representation of the reality (the task of sciences) is based, accordingly to Wittgenstein, in the theorem (1.1) of the *Tractatus*: the world is constituted by the totality of facts, not of things (Theorem 1.1). The fact, if it is the case, exist as an elementary (atomic) fact (1.21-2), resulting from a (minimal) particular combination of objects (2.01). Objects are simple (2.02), elementary, fixed (2.026) entities. But the *objects by themselves, outside facts, are only possibilities of facts*, nothing in reality (2.011) except their forms, qualities to be part of facts (2.0141). These qualities determine the possibility of facts (2.012): “objects contain the possibility of all states of affairs” (2.014). The object is the fixed, the existent; the configuration is the changing, the variable (2.0271). In the elementary fact objects hang one in another, like the links of a chain (2.03), combined in a definite way (2.031). The way in which objects hang together in the fact is the structure of the fact (2.032). And finally: the form (of objects) is the possibility of the structure (2.033).

Examining these Wittgenstein's theorems, a microbiologist will immediately be attracted and even moved by the idea that the philosopher is speaking about life, with all its unveiled evolutionary possibilities, based on alternative molecular configurations that give rise to different facts, a game in which molecules themselves are nothing: the *objects by themselves, outside facts, are only possibilities of facts*. If Biology is the art and science of *representing* life, such representation should be sufficiently faithful to reflect the complexity and the dynamics of facts in the unthinkable real life, and, understanding the links in the representation (the model) we should assume that something “similar” should occur, at least in part, in the true life.

## The effects of representations from zeuxis to schubert

In the fifth century BC, Zeuxis depicted the grapes so realistically that birds flew down to peck them: *Ars simiae Naturae*. In this example, grapes are considered by birds to be alive, so that the representation not only faithfully corresponds to life, but produces the *same effects*. Modifying in the model (the picture) the shape or color of the grapes, the birds will not be attracted anymore, so that we could presume which is the attractive properties of real grapes. Of course the result of the experiment can be wrong. For instance, birds could be attracted in the picture by the odor of oil or egg used as a solvent of a particular dry used to paint the grapes, and not by its realistic color. But the experiment might also be true, and the birds could effectively be attracted by the painted grapes. The representation has an *effect*, which might be similar, or even identical, to the effect caused by what is represented. Now let us birds to examine for a little longer the famous Zeuxis's grapes. Certainly they will be soon disappointed, and if challenged again by the image, they will not be attracted any more. As in the famous Türing metaphor, life will recognize life, provided a certain period of examination.

The idea of “music of life” has been developed recently by the famous physiologist Dennis Noble (Noble, 2006). The authors of the present essay were simultaneously disappointed and flattered when he found that Noble used in his book “The Music of Life” almost exactly the same example that we also used in the first version of our manuscript, produced years ago, namely the Schubert's Piano Trio in E flat major, D.929; the author's choice was its ancestor, the Piano Trio in B flat, D.898. Noble even considered the space travelers metaphor, even though not entirely in our way. The important thing is that all of us were *impressed* emotionally by that piece of music. “As the music entered into the slow movement,” “I cried” confess Noble. Beyond any possible doubt, music produces *experiences* and *effects*. If music were an unthinkable representation of life, we are experiencing effects because this representation provides an obscure perception of the *will* of life, in the Schopenhauer sense. But note that if this perception will be very difficult or impossible to obtain just looking at the score, or the irregularities of the grooves of a gramophone record.

## Half-thinking, half-seeing

Ludwig Wittgenstein evolved in his posthumously published book “Philosophical Investigations” (Wittgenstein, 1953; McGuinn, 1997) to the proposal of some possible ways for understanding the realities that can be *shown*, but not *thought*, or at least, not entirely thought. The experience of seeing something is converted in a perceptual experience, and thus regarded as indistinguishable from a thought (Bar-Elli, 2006). As Wittgenstein says, “-is it a case of seeing and thinking? Or an amalgam of the two, as I should almost like to say?” (PI 197). Let us imagine a complex feature that can be only shown, as a human face. The image of the face depends of a huge network of anatomical interactions involving the shape of bones, the volume of the muscles, the amount and distribution of subcutaneous fat, and many other factors. But also these features reflect the age, the sex, ethnicity, the diet, or even the character of the underlying human being, so that the looking at this face might produce *effect*s. A full description of the dynamic network of elements giving rise to a particular recognizable face will be almost impossible. Of course, rough approximations might be attempted, as anthropologists trying to reconstruct from the bones and the presumed diet the face of Lucy, our hominid ancestor. But the example that we are discussing here is that the aspect of the face that we *see* is “condensed information” of a complex network of elementary facts. Even more, without need of knowing almost anything of the generational network of interactions giving rise to the face, the face can be remembered and *compared* with other faces at an extremely specific level of discrimination. The person recognizing a face has an “experience of meaning.” As the face might reveal family resemblances, Wittgenstein suggests a kinship between *seeing* an aspect and the *experience of meaning*. The experience of meaning is half-thinking (Bar-Elli, 2006).

Of course the musical metaphor is exploited by the later Wittgenstein of “Last writings on the Philosophy of Psychology” in an identical sense (Wittgenstein, 1949–1951; Worth, 1997). Music results from a complex dynamic interplay of elements, has not a clear semantic structure and, as the face, produces effects, an *experience of meaning* that enable *connections* and *comparisons*. For instance, he says about a musical theme: “I could compare it with something else which has the same rhythm (I mean *the same pattern*)” (I.382). The understanding of a musical theme is based on the experience of what he defines as “internal relations” occurring in the otherwise “unthinkable” musical stuff. Bar-Elli has pointed out the critical importance of the concept of the *experience of meaning* in Wittgenstein as a part of a synoptic view (*übersicht*) of understanding (Bar-Elli, 2006). Music is an excellent equivalent of life in terms of exploring intelligibility of complex systems, probably superior to language, precisely because we seem to lack here any grip on an idea of semantic units, which is so often conceived as the basis of linguistic analogy. As the book of music cannot be reduced to the music score, and then music is music, “my central argument is that the book of life is life itself” says Dennis Noble (Noble, 2006). In conclusion, our understanding of complex systems as life or music depends *both* on: (1) the understanding of their representations (as genomes or scores), and (2) the understanding (under the form of experience of meaning) of something that can be only seen or show, but still compared or connected. Half-thinking, half-seeing: the HT-HS strategy.

## Implementing the HT-HS strategy: complex models of complex systems

How the HT-HS, half-thinking, half-seeing strategy could be applied to increase the intelligibility of complex biological systems, and life in general? For the half-thinking part, it is obvious that we should maintain a high-level descriptive research as it is being done in genomics, proteomics, metabolomics, or transcriptomics of particular organisms, complemented by the dynamic approach provided by fluxomics, all within the frame of more and more computationally advanced systems biology. All that is research on the composition of Wittgensteinian *atomic facts* and *propositions*; of course that includes certain level of synthesis, what one of us (Moya et al., 2009) proposed to call *synthetic view one*. This level is the level of anatomy and physiology, or, in linguistic terms, *legibility*. All these approaches essentially would serve to provide material (organized material) to feed complex models, able to move into the *synthetic view two* (Moya et al., 2009). Possibly the advances in Systems Biology will provide useful integrative models, but they will be insufficient to provide by themselves full intelligibility of functional processes. Brenner contend that this approach is insufficient, as deducing models of function from the behavior of a complex system is an inverse problem that is impossible to solve (Tarantola, 2006; Brenner, 2010).

The half-seeing part might start when we could be able of developing more powerful model tools to run simultaneously all data and processes generated by these—omics, in a comprehensive and integrated way. For that a purpose we need “Big Science,” based on the convergent interactions among scientists of many disciplines, and not only from biology (Nurse, 2008). If we were able to represent the holistic result of such synthetic approach, we will be near something as a complex image of a living structure, able to be seen or to be showed. And more importantly, able to be “physiognomically” compared and related with other images obtained from other organisms. At this stage we should reach *imageability*. The more advanced part of the half-seeing part should be based on multi-hierarchical understanding of life, and the expected appearance of *emergent* qualities, particularly if *communication* strategies between levels are assured.

Modeling trans-hierarchical complex levels is certainly one of the biggest challenges we have (Campbell, 1974; Martínez and Moya, 2011). These models would eventually provide different levels of predictability. Indeed predictability is the best touchstone to validate the reality of complex models for complex systems (Martínez et al., 2007). Maybe even complex models might be able to predict just the next steps of biological processes, and only in close space and time compartments, just as meteorological predictions based on cumulative empirical observations. Also, and as we mentioned before (at least for non-atonal music) the experience allows to predict the evolution of a melody, at least for a few compasses. In any case, complex models will serve to continuously provide material to be tested and rejected when not mating with reality (Brenner, 2010). We should in any case be aware that to validate models by comparing them with “reality” might be a circular problem, as the “reality” could be only defined by models. That is why we should be able to “see” the reality, even in an obscure, fuzzy way. Paraphrasing Albert Einstein (Einstein, 1944; Dougherty and Bittner, 2011) the propositions, the rationality of the models (half-thinking) should be “firmly connected with sensory experiences” (half-seeing).

As Denis Noble says, life should be considered in a variety of levels; life is “a kind of music, a symphonic interplay between genes, cells, organs, body, and environment,” what can be only examined under the views of synthetic biology (Noble, 2006). Microbiologists are among the best placed scientists to mature these concepts, as they have daily experience of the complex interplay of genetic sequences and domains, operons, genes, proteins, macromolecular complexes, signaling networks, adaptive and regulatory functions, different classes of nested mobile genetic elements, clones, species, communities, integrated microbiotic ensembles, and microbial ecology at large. We have to deal with huge diversity of *facts* or *pieces*, constantly offering in a trans-hierarchical way new complex patterns to evolutionary processes (Baquero, 2004, 2009, 2011; Wagner, 2012a,b). In short, we hope that the future will allow scientists to cover the three phases of this epistemological process will be: legibility, imageability, and intelligibility of complex biological systems. Mixing half-thinking and half-seeing, the scientific method that might be we should apply to understand the complexities of microbial life.

### Conflict of interest statement

The authors declare that the research was conducted in the absence of any commercial or financial relationships that could be construed as a potential conflict of interest.
